# MOSAIC: A cohort study of human mpox virus disease

**DOI:** 10.12688/wellcomeopenres.19616.2

**Published:** 2023-10-27

**Authors:** Elise Pesonel, Isabelle Hoffmann, Laetitia Guiraud, Josephine Bourner, Alpha Diallo, Jake Dunning, Peter Horby, Sabrina Kali, Cédric Laouénan, France Mentré, Laura Merson, Diana Molino, Romain Palich, Amanda Rojek, Evelina Tacconelli, Coralie Tardivon, MOSAIC study group, Yazdan Yazdanpanah, Alexandra Calmy, F-Xavier Lescure, Piero Olliaro

**Affiliations:** 1 ISARIC - Pandemic Sciences Institute, University of Oxford, Oxford, UK; 2 AP-HP. Nord, Hôpital Bichat, Department of Epidemiology Biostatistics and Clinical Research, Paris, France; 3 HIV/AIDS Unit, Division of Infectious Diseases, Geneva University Hospitals, Geneva, Switzerland; 4 INSERM-ANRS| MIE, France, Paris, France; 5 Université Paris Cité, INSERM, IAME UMR 1137, Paris, France; 6 Sorbonne University, Infectious Diseases department, Pitié-Salpêtrière hospital, AP-HP, Pierre Louis Epidemiology and Public Health institute (iPLESP), INSERM U1136, Paris, France; 7 University of Verona, Verona, Italy; 8 AP-HP Nord, Hôpital Bichat, Department of Infectious and Tropical Diseases, Paris, France

**Keywords:** Mpox, observational study, low-intervention clinical trial, tecovirimat

## Abstract

**Background:**

Human mpox is a viral disease caused by an Orthopoxvirus, human mpox virus (hMPXV), typically causing fever and a rash. Mpox has historically been endemic to parts of Central and West Africa, with small numbers of imported cases reported elsewhere, but starting May 2022 an unprecedented global outbreak caused by clade IIb hMPXV was reported outside traditionally endemic countries. This prompted the initiation of MOSAIC, a cohort study implemented in Europe and Asia that aims to describe clinical and virologic outcomes of PCR-confirmed hMPXV disease, including those who receive antiviral therapy. The study is ongoing.

**Methods:**

MOSAIC recruits participants of any age with laboratory-confirmed mpox disease who provide informed consent. Participants enrol in the cohort for a total of six months. Blood, lesion and throat samples are collected at several timepoints from the day of diagnosis or the first day of treatment (Day 1) until Day 28 for PCR detection of hMPXV. Clinical data are collected by clinicians and participants ( *via* a self-completion questionnaire) for six months to characterize the signs and symptoms associated with the illness, as well as short- and more long-term outcomes.

**Discussion:**

The design and prompt implementation of clinical research response is key in addressing emerging outbreaks. MOSAIC began enrolment within two months of the start of the international mpox epidemic. While the number of cases is now low, the study remains open for inclusion.

**ICTRP registration:**

EU CT number: 2022-501132-42-00 (22/06/2022)

## Introduction

### Background and rationale

Mpox (formerly Monkeypox) is an acute viral disease caused by the human mpox virus (hMPXV), a member of the Orthopoxvirus family for which there are two distinct genetic clades, Clade I and Clade II ^1, 2^. Clade I is associated with higher morbidity and a case fatality rate of approximately 11% ^3^ and circulates primarily in parts of Central Africa. Clade II consists of two sub-clades–Clade IIa, historically detected in parts of West Africa (case fatality rate 0–4% ^3^), and Clade IIb, identified during the global outbreak starting in May 2022–sometimes also referred to as clade III ^4^.

The typical clinical features of human mpox closely resemble those of smallpox, although mpox is considered to be a milder disease. Clinical features of mpox are highly variable, but typically include distinctive centrifugal skin lesions, lymphadenopathy (which is not present in smallpox), fever, and other non-specific symptoms. However, in the 2022 clade IIb outbreaks, which mostly affected men who have sex with men ^5^, the clinical presentations differed from those described for cases in previous mpox outbreaks elsewhere. For example, 2022 clade IIb outbreak cases often presented with systemic symptoms typical for mpox, but with highly regional and sometimes more severe lesions, including oropharyngeal, genital, peroneal (peri-anal and ano-rectal lesions), frequently associated with other sexually transmitted diseases. Additionally, the incubation period was shorter (nine days compared to 13 days; range three to 34 days) for clade IIb outbreak cases, and the case fatality rate (CFR) was lower ( <0.01%) compared with the CFR previously reported for Clade II outbreaks in Africa [1.7–6.8%] ^3^.

The antiviral agent tecovirimat is the only mpox treatment licensed recently in Europe by the European Medicines Agency (EMA, in January 2022) and by the UK Medicine and Healthcare products Regulatory Agency (MHRA, in July 2022). Approval was granted under ‘exceptional circumstances’, based on experimental and human safety data, but no clinical efficacy trial had been performed at that time. Tecovirimat, and the unrelated antiviral brincidofovir, are licenced for treatment of smallpox by US Food and Drug Administration (FDA) under the ‘animal rule’.

The unprecedented 2022–23 international outbreak of clade IIb mpox is associated with over 91,000 cases reported in non-endemic countries from May 2022 and till present, of which around 26,000 confirmed cases have been reported in Europe and around 900 in Asia.

In response to the outbreak, the MOSAIC study was conceptualized on 20 May 2022, following a request from the EMA’s Emergency Task Force (ETF). MOSAIC was influenced by experience accrued through involvement in the ongoing tecovirimat expanded access programme (EAP) in the Central African Republic (CAR) ^6^, and selection of appropriate endpoints was aided by collaboration with researchers working on other studies, such as the PALM-007 study in the Democratic Republic of the Congo. Here we describe the design of the ongoing MOSAIC study, progress to date, and some of the challenges experienced in executing the study.

This protocol has been written according to the SPIRIT guidelines ^7^.

## Methods

### Objectives

The primary objective of this study is to describe clinical outcomes of participants with laboratory confirmed MPXV, whatever the treatment administered.

Secondary objectives describe the virologic evolution of mpox, and Adverse Events (AEs) and Serious Adverse Events (SAEs) that occur in participants enrolled in the study. In France, additional exploratory objectives aim to evaluate virologic and immunological outcomes to learn more about viral transmission and induced immune responses. Among a subset of participants, exploratory objectives are to determine: i) the distribution of MPXV within the different anatomical sites of the participants, as well as the duration of positivity of the samples at the different sites, and the viral dynamics in patients receiving or not antiviral treatments (including tecovirimat), ii) the viability of the viruses detected by PCR (cell culture), iii) the intra-individual and inter-individual genetic variability, iv) the potential resumption of human immunodeficiency virus (HIV) viral replication in the blood and semen of participants living with HIV and the presence of other sexually transmitted infections, and v) post-mpox acquired humoral and cellular immunity.

### Trial design

MOSAIC is a multi-centre, multi-country cohort study enrolling participants with laboratory confirmed mpox for six months’ follow up. The study is an observational study in participating non-European Union (EU) countries, but has been classified as a Low Intervention Clinical Trial (LICT) in the EU–in accordance with the EU Clinical Trial Regulation (CTR) 536/2014 ^8^, MOSAIC fulfils a LICT conditions as an authorised medicinal product, tecovirimat, is being used in compliance with its marketing authorisation ( *e.g.*, its use is not influenced by this protocol) and the study procedures do not pose more than minimal additional risk to the safety of its participants.

**Figure 1.**
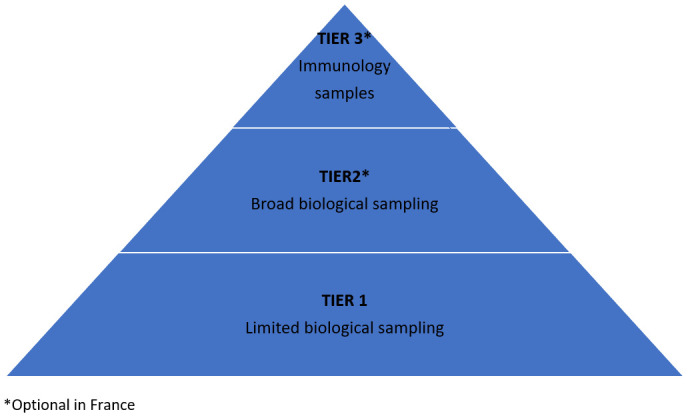
Tiered approach.

The study is designed with a tiered approach to protocol activities ( Figure 1 and Table 1). Tier 1 activities–characterised by limited biological sampling and data collection–apply to all participants enrolled in the study. Tiers 2 and 3 apply only in France where participants are invited to give consent for additional, optional, activities associated with these tiers. Under these tiers, additional virologic (Tier 2) and immunological (Tier 3) investigations are carried out.

**Table 1.** Schedule of procedures.

TIER				Baseline ^a^	Study days or Treatment days (D)																	
					D1	D2	D3	D4	D5	D6	D7	D8	D9	D10	D11	D12	D13	D14	D21	D28	D60	D180
**1**	**2**	**3**	*Windows permitted*															*±2 days*		*±2 days*	*±7 days*	*±7 days*
x	x	x	**Baseline assessment**																			
x	x	x	Consent	**X**																		
x	x	x	Demographics	**X**																		
x	x	x	Medical history ^b^	**X**																		
x	x	x	**Medical assessment**	**X**	**X**	*For patients who are hospitalised ^c^ or if a patient attends a pre-* *scheduled clinic visit*												**X**		**X**	X	X
x	x	x				X	X	X	X	X	X	X	X	X	X	X	X					
x	x	x	**Outcome assessment**															**X**		**X**	X	
x	x	x	**Sampling** ^d^																			
x	x	x	Two lesion swabs	**X**				X				X						**X**	X ^e^	**X**		
x	x	x	Throat swab	**X**				X				X						**X**	X ^e^	**X**		
	x	x	Mucosal lesion swab ^f,g^	X																		
	x	x	Saliva sample (using swab) ^f^	X								X						X		X	X ^h^	X ^i^
	x	x	Anus swab ^f^	X								X						X		X	X ^h^	X ^i^
x	x	x	Blood sample collection ^e^	**X**				X				X						**X**	X ^e^	**X**		
	x	x	Supplementary blood sample for serum	X				X				X						X		X	X	X
		x	Supplementary blood sample for plasma																	X	X	X
	x	x	Urine sample	X								X						X		X	X ^h^	X ^i^
	x	x	Semen or vagina secretions sample	X								X						X		X	X ^h^	X ^i^
x	x	x	**Participant self-assessment survey**	X	X	X	X	X	X	X	X	X	X	X	X	X	X	X	X	X	X	X
	x	x	**Participant social and sexual** **behaviour self-questionnaire**	X																		

*Note:* X in bold are data collection and sampling timepoint to prioritise.

^a^Baseline (could be anytime from D1) – baseline assessment and baseline medical assessment are done at enrolment; baseline samples are the participant’s diagnostic samples, which may have been collected for routine care before study participation commenced.

^b^Includes comorbidities, results of pregnancy and human immunodeficiency virus (HIV) tests, data on signs and symptoms experienced by the participant since their mpox diagnostic sample was collected, and details of potential adverse events (AEs) and serious adverse events (SAEs) occurring since their mpox diagnostic sample was collected.

^c^For hospitalised participants, hospital assessments should continue daily until the point of discharge

^d^Samples are only collected where feasible and permitted under local and national regulations on mpox infection prevention and control. Where local capacity limits sample collection, sites should prioritise the collection of D14 and D28 samples—all other samples are optional. Baseline samples are the participant’s diagnostic samples, which may have been collected for routine care before study participation commenced.

^e^An additional sample should be collected at D21 if the participant has a positive PCR on D14.

### Outcomes

***Primary outcome measure.*** The primary outcome measure of this study is the participant’s time to lesion resolution without any serious complications. Lesion resolution is the first day on which all lesions are resorbed, scabbed or desquamated and mucosal ulcers healed. Lesion resolution is assessed until a maximum of 14 days since the date that the diagnostic sample is taken (for participants who do not receive mpox-specific antiviral treatment) or the day of treatment initiation (for participants who receive mpox-specific antiviral treatment).

***Secondary outcomes.*** Secondary outcome measures evaluate clinical and virologic outcomes. Clinical outcomes are assessed on the basis of:

1. Clinical status on day 14 and day 28 according to a four-point ordinal scale assessed by a physician or a study nurse. The ordinal scale is a) all lesions are resolved and no serious complications, b) active lesions and no serious complications, c) serious complication and/or hospitalization due to mpox, or d) death.
2. Evidence of recrudescence or relapse at day 60 and day 180.

*Note:* A "serious complication" is any complication associated with a Serious Advent Event (SAE) that is life-threatening, results in hospitalisation, prolongation of existing hospitalisation, disability, incapacity, or a congenital anomaly; or any other complication that is considered medically significant.

Virologic outcomes in Tier 1 are assessed on the basis of:

1. Change from baseline in mpox virus DNA levels in throat swabs on days 4, 8, 14 and 28.
2. Change from baseline in mpox virus DNA levels in blood on days 4, 8, 14 and 28.
3. Presence of mpox virus DNA in lesion swabs on days 4, 8, 14 and 28.

The study also assesses the number and type of SAEs, Suspected Adverse Reactions (SARS) and Suspected Unexpected Serious Adverse Reactions (SUSARs) within 28 days of enrolment.

Pregnancy outcomes defined as the final known health status of the mother and child are evaluated in any participants who are pregnant during the course of the study.

***Exploratory outcomes in Tier 2 and Tier 3.*** Further virologic and immunological outcomes are also evaluated in France under this protocol to learn more about viral transmission, humoral and cellular immune responses. The outcome measures are as follows:

- Detection and quantification of MPXV using different PCR tools from different body samples (skin lesions, mucosal lesions, blood, urine, genital secretions, oropharynx, saliva, anus) at baseline, D8, D14 and D28. Additional samples are collected and tested at D60 and D180 if participants receive a positive result at D28 or D60, respectively;
- Capacity of MPXV to infect cells, and replicate, investigated by cell culture and MPXV detection from supernatant at baseline;
- Genetic diversity of MPXV between the participants and between the different anatomical sites, assessed with MPXV deep sequencing;
- Detection of syphilis using serological test, and other sexually transmitted infections (STIs) ( *Chlamydia trachomatis*, *Neisseria gonorrhoeae*, *Mycoplasma genitalium*) using PCR from urine, anus and oropharynx, at baseline;
- Quantification of HIV viral load from blood and semen in participants living with HIV at baseline, D8, D14 and D28;
- Description of social and sexual behaviours in the months prior to inclusion;
- Quantification of plasma antiviral concentrations;
- Assessment of the diversity of genetic immune host factors;
- Quantification and dynamic of the humoral and neutralizing memory B cell responses, as well as the cellular T cell response, over time, at baseline, D14, D28, D60 and D180.

### Sample size

As this study is being conducted in the context of an outbreak, in which the study aims to generate as much information as possible about a disease that has previously been poorly characterised, no target sample size has been pre-defined for the purpose of the analysis, which is primarily descriptive.

However, in order to satisfy regulatory requirements, the study has been approved to enrol a maximum of 1,400 participants.

### Study setting

Participants are enrolled in secondary care settings across Europe and in Singapore, and may be managed as either inpatients or outpatients. Other countries may be added to the study as the outbreak evolves.

### Eligibility criteria

***Inclusion criteria***

1. Patients with:
  a. Laboratory confirmed mpox virus disease
  b. Laboratory confirmation pending, but who are being managed as a presumptive case

2. Informed consent provided for participation in the study

A presumptive case is defined as a patient who is managed as a case of mpox due to high clinical suspicion of disease (according to the prevailing World Health Organization (WHO) case definition) or high risk of serious disease (such as immunosuppression). A patient who is a presumptive case and subsequently tests negative for MPXV exits the study.

No exemptions are made regarding eligibility. Each participant must satisfy all the approved eligibility criteria.

***Exclusion criteria***

1. Presumptive cases with a subsequent negative test for MPXV
2. Adults lacking capacity due to a previously diagnosed impairing condition
3. In Tier 2 and Tier 3 in France only: participants under the age of 18 years
4. In Singapore only: participants under the age of 21 years and adults who lack capacity to provide written informed consent for themselves

### Recruitment

Participants are enrolled in the study before laboratory confirmation of mpox on the basis of high clinical suspicion or at any time after they receive a positive diagnosis of mpox infection by PCR. From June 2022 to December 2022, new cases were recruited prospectively from participating sites that had been activated. Then, from January 2023, as new cases were rare, patients managed before the start of the study at participating sites or patients from newly activated sites were recruited retrospectively.

Potential participants are identified and approached to participate in this study by their clinical care team at a participating site. Identification may take place using the Site Investigator’s patient register. The Site Investigator must, in all cases, confirm that the potential participant meets the eligibility criteria before proceeding further with the inclusion process. Any patient meeting the inclusion criteria can be enrolled in the study whether they are currently managed or have previously been managed as a mpox case, provided they give their informed consent to participate.

If the potential participant meets all the eligibility criteria, information about the study is provided using the participant information sheet. The participant information sheet can be given in person during a clinic visit, or in the event that the patient is currently being managed as an outpatient and has no upcoming scheduled clinical visit, the information sheet can be sent by post or email, or it can be explained by telephone (according to the site’s locally approved practices).

The participant information sheet details no less than: the nature of the study; what it involves for the participant; the implications and constraints of the protocol; and any risks involved in taking part. It is clearly stated that the participant is free to withdraw from the study at any time for any reason without prejudice to future care, without affecting their legal rights or their care and with no obligation to give the reason for withdrawal.

The participant is permitted as much time as desired to consider the information, and given the opportunity to question the Investigator, their doctor or other independent parties to decide whether they participate in the study.

Participation is entirely voluntary.

### Who takes informed consent?

Consent is taken by the patient’s clinical management team based at the health facility in which their mpox illness is currently or was formerly being managed.

The participant enrolled during a clinic visit must personally sign and date the latest approved version of the Informed Consent form before any study specific procedures are performed.

For participants who are being managed as outpatients and do not have a scheduled clinic visit, consent can be obtained by phone, post or e-signature (according to the recruiting site’s local practices and approvals).

The person who obtained the participant’s consent in person or remotely must be suitably qualified and experienced, and have been authorised to do so by the Principal Investigator (PI). A copy of the signed informed consent form is given to the participant. An original signed form is retained at the study site.

In the UK, at sites participating in both MOSAIC and the UK Clinical Characterisation Protocol (UK CCP), participants can be co-enrolled in both studies.

Specific document and consent procedures are in place for consent of vulnerable population. Please see ‘Additional File 2: Appendix B’ in *Extended data* ^7^ for the consent process for children, participants who are unable to write, and adults who lack capacity to provide consent for themselves.

At the start of the study, consent was not required in France. Pending the EU approval, the French Ethic Committee authorised the study on 27 June 2022 as an observational study or “category 3” study according to the ‘Jardé Law’ for which in accordance with Article L 1122-1 of the Public Health Code, patients may be recruited into the study for data collection only after they have been informed of the purpose, nature, constraints and foreseeable risks of the study and have not objected to the use of their data in this context. Only the investigator must sign and date a no opposition form. On the 17 August 2022, the EU approved the study as a low-intervention clinical trial, so from this date, the study in France follows European requirements and requires signed consent as described in section *Recruitment* in this protocol.

Please see ‘Additional File 2: Appendix D’ in *Extended data* for details about taking remote consent in the UK (including phone consent) ^7^.

### Additional consent provisions for collection and use of participant data and biological specimens

Optional consent for the use of anonymised samples collected under MOSAIC in future ethically approved research is offered to all participants enrolled in the study.

In France, participants can enrol in Tiers 2 and/or 3 of the study, which explore further virologic and immunological outcomes. A separate participant information sheet and consent form are provided for participants to consider and provide optional consent for these additional study procedures.

### Interventions

This is an observational study. Patients receive standard care as deemed appropriate by the treating physician and depending on availability of approved medications.

### Explanation for the choice of comparators

This is an observational study, there is no control arm.

### Criteria for discontinuing or modifying allocated interventions for a given trial participant

All treatment decisions for discontinuing or modifying treatment are made at the discretion of the patient’s treating physician.

### Strategies to improve adherence to intervention protocols, and any procedures for monitoring adherence

Treatment adherence is monitored by the treating physician as part of routine care.

### Relevant concomitant care and interventions

All concomitant care is given at the discretion of the treating physician.

### Sequence generation

This is not a randomised clinical trial and participants are not randomised to treatment.

### Allocation concealment mechanism

This is not a randomised clinical trial and participants are not randomised to treatment.

### Implementation

This is not a randomised clinical trial. Participants are not randomised to a treatment given at the discretion of the treating physician.

### Blinding (masking)

This is not a blinded study and therefore no-code breaking procedures applies.

### Provisions post-trial care

If a participant suffers harm as a result of their involvement in the study, the Sponsor has a specialist insurance policy in place that would operate in the event of any participant suffering harm as a result of their involvement in the research (Newline Underwriting Management Ltd, at Lloyd’s of London).

### Participant timelime

Enrolment is the day that the informed consent form is signed, and the participant is enrolled in the study. Enrolment may be any day once the diagnostic sample is taken or after the patient has been considered a “presumptive case” by their treating clinician.

After enrolment, days are counted from the date the diagnostic sample is taken:

- Day 1 is the date that the first sample to diagnose mpox was taken, regardless of when results became available. If the participant is enrolled on the same day that the diagnostic sample is taken, enrolment occurs on Day 1.
- If a participant is enrolled after the date that the diagnostic sample was taken, then study procedures start from the number of days since the sample was taken.

Participants who do not receive treatment follow the schedule from D1 and continue until D180. Participants who receive treatment immediately following diagnosis and enrolment in the study follow the schedule described above.

If a participant enters the study without receiving treatment and then begins receiving treatment at a later date, they initiate a new schedule of procedures by restarting at D1. This second D1 represents the date of treatment start. This extends the study participation period by the number of days between enrolment and treatment initiation.

### Data collection methods

All data are recorded by the clinical and study teams directly on the REDCap (version 13.1.30) ^9^ electronic Case Report Form (eCRF) for each timepoint as defined in Table 1.

***Demographics and medical history.*** Data relating to the participant’s demographics and medical history are collected at baseline. Demographic includes sex at birth and ethnic group. Medical history includes data on comorbidities, results of pregnancy and HIV tests, data on signs and symptoms experienced by the participant between the collection of mpox diagnostic sample and the point of consent, and details of potential AEs and SAEs occurring between the collection of mpox diagnostic sample and the point of consent.

***Medical assessments.*** Medical assessments are undertaken at baseline, and D14, 28, 60, and 180. These assessments can be conducted in person, *via* video link or telephone. The data collected include the participant’s signs and symptoms, any treatment they may be receiving for mpox, AEs and SAEs details.

***Biologic sampling.*** Baseline samples are the participant’s diagnostic samples that may have been collected under routine care before the participant signed the consent form. Blood* (~0.5ml to 20ml depending on local collection procedures), two lesion swabs and one throat swab are collected at D4*, 8*, 14, 21* and 28 where feasible to do so and permitted under local and national regulations–the asterisk (*) denotes procedures only for hospitalised subject; day 21 and 28 sampling only if day 14 sample is still positive.

Where local capacity limits sample collection, sites should prioritise the collection of D14 and 28 samples. All samples are tested by PCR only conducted in local laboratories in each participating country.

In Tier 2 in France, in addition to the sampling covered in Tier 1, additional samples of mucosa, saliva, anus, urine, semen or vaginal secretions, and blood are collected at baseline, D8, D14, D28, D60 (in case of oral, genital or anal lesion) and D180 (only if sample is positive at D60).

In Tier 3 in France, in addition to the sampling covered in Tier 1 and 2, additional samples of blood are collected at D28, D60 and D180.

Samples in Tier 2 and 3 are tested as in Table 2.

**Table 2.** Tests by sample type.

TIER	Test	Sample							
		Lesion	Throat	Mucosal lesion	Saliva	Anus	Blood	Urine	Semen or vagina secretion
1	PCR	X	X				X		
1&2	Bacterial STIs detection		X			X	X	X	
1&2	HIV-RNA quantification						X		X
1&2	MPXV quantification (sPCR) + infectivity assessment (cell culture)	X	X	X	X	X	X	X	X
1&2	MPXV sequencing	X	X	X	X	X	X	X	X
1&2	Humoral immune response characterisation (anti- MPXV antibodies quantification, seroneutralization)						X		
1&2 &3	Cellular immune response characterisation						X		
1&2	Genetic host factors determination + auto-immune antibodies detection						X		
1&2	Tecovirimat pharmacokinetics						X		

HIV, human immunodeficiency virus; MPXV, mpox virus.

Please refer to ‘Additional File 2: Appendix F’ in *Extended data* for further information on sample processing, analysis and storage in the UK ^7^.

***Participant self-assessment survey.*** All participants are asked to complete a self-assessment survey from the point of entry into the study until D180 *via* a secure online questionnaire. The questionnaire, completed *via* online REDCap survey, captures information about the participant’s signs and symptoms, including lesion status.

For Tier 2 and Tier 3 in France, the baseline self-assessment survey includes an additional social and sexual behaviour questionnaire.

***Outcome assessment.*** During the medical assessment on D14, 28, 60 and 180, the clinical status according to a four-point ordinal scale (see the *Outcomes* section) is recorded.

For participants who are pregnant at any time during the study, they are followed in the study until their pregnancy outcome is known.

*Note:* As participants can consent to enrol in the study at any point after receipt of a positive PCR result for mpox infection, the eCRF can be completed using existing medical notes and results of samples already taken.

### Plans for collection, laboratory evaluation and storage of biological specimens for genetic or molecular analysis in this trial/future use

There are no plans to conduct genetic or molecular analysis on biological samples collected from participants enrolled in this study.

Participants have the option to consent to the use of their biological samples for future research. Future research undertaken on these samples will undergo relevant reviews by the responsible national ethics committees and/or institutional review boards before any further analyses are undertaken.

### Plans to promote participant retention and complete follow-up

The study has a flexible approach to data collection and sampling to reduce the research burden on both participants and staff who are working in the context of an outbreak.

Table 1 describes the flexibility in the days on which samples and assessments can be undertaken.

Equally, samples should only be collected where it is feasible to do so and priority samples are identified in the *Data collection methods* section above.

Research staff at participating sites are asked to make every reasonable effort to follow participants throughout the entire study period.

### Early discontinuation and withdrawal of participants

Throughout the duration of the study, participants have the option to withdraw prematurely for various reasons, including but not limited to:

- If a presumptive case enrolled in the study tests negative for MPXV, they are withdrawn.
- Participants may perceive certain adverse events (AE) as intolerable, leading to their withdrawal.
- Inability to adhere to the study procedures can also be a reason for withdrawal.
- Participants have the autonomy to decide to withdraw from the study.
- In some cases, the clinician may make the decision to withdraw a participant.

The study design provides participants with three withdrawal options, which are as follows:

1/ Participants can choose to withdraw from active follow-up and further communication. However, they allow their direct care team, acting on behalf of the study, to continue accessing their medical records and relevant hospital data that is routinely recorded as part of standard care. This data is essential for data collection on the electronic case report form (eCRF).

2/ Participants have the choice to withdraw from the study while permitting the retention of data and samples obtained up until the point of withdrawal. These retained data and samples will be used for analysis in the study, but no additional data or samples will be collected after the withdrawal.

3/ Participants may opt to completely withdraw from the study, including the request to remove the data and samples collected up until the point of withdrawal. In this case, the data and samples already obtained will not be utilized in the final study analysis.

The eCRF records both the type of withdrawal and the reason for withdrawal, ensuring proper documentation and tracking of participant decisions and the underlying motivations for their withdrawal from the study.

### Expenses and benefits

There is no cost to participants and no payment is made for participation in the study. Participants are reimbursed for incidental expenses related to enrolment according to local approvals and practice.

### Definition of end of study

End of study is defined as the date of the last visit (D180 or date pregnancy outcome is known) of the last participant enrolled in the study or the date that all samples have been analysed, whichever is later.

There are jurisdictional differences in reporting end of study data. In the EU, a summary of study results will be submitted within one year of the last participant visit and will not be postponed for secondary sample analysis. This information will be provided without delay when they are available.

## Data management

### Source data

Source documents serve as the initial records of data and are used to obtain participants' data for the electronic case report form (eCRF). These source documents encompass a variety of materials such as hospital records (including medical history and medication details summarized into the eCRF), clinical and office charts, laboratory and pharmacy records, diaries, microfiches, radiographs, and correspondence. If the eCRF is the sole place where data is originally recorded without any other written or electronic records, the entries in the eCRF are considered source data.

In this study, primary data collection occurs through two main methods:

(i) Study staff at participating sites complete electronic case report forms on enrolment, during scheduled study visits, daily for in-patients, and whenever laboratory results become available.

(ii) Participants complete an online questionnaire daily during the first 14 days of the study (or while they are in the hospital) and at specific time points (D21, D28, D60, and D180).

The data entered in the electronic case report forms and the online questionnaire are stored in separate REDCap databases.

To ensure data quality, automatic data checks are programmed into the REDCap systems, alerting users during data entry to minimize missing, inconsistent, or erroneous data. Study sites are responsible for reviewing the data entered into the case report forms for their respective participants.

Centralized monitoring of all data collected in the study is conducted by the University of Oxford. This monitoring involves evaluating data compliance with the study protocol and assessing accuracy, as outlined in the study-specific Monitoring Plan. When issues are identified, queries are triggered to be resolved by the respective sites. If any issues are detected in the Participant Self-Assessment data, the study site is notified to communicate with the participant if necessary.

On-site monitoring may be necessary if serious or repeated non-compliance with the protocol is identified through centralized monitoring. Local or regional coordinating centers will carry out this on-site monitoring.

The REDCap database is hosted at the University of Oxford for a minimum of 25 years following completion of the study.

### Confidentiality

***Participant confidentiality.*** The study complies with the UK General Data Protection Regulation (UK GDPR), Data Protection Act 2018 GDPR (EU) 2016/679 and the Personal Data Protection Act (Singapore), which require data to be de-identified as soon as it is practical to do so. The processing of the personal data of participants is minimised by making use of a unique participant study number only on all study documents and any electronic database(s). All documents are stored securely and only accessible by study staff and authorised personnel. The study staff safeguards the privacy of participants’ personal data.

All documents and samples are labelled with a pseudonymised subject ID code. Identifying information collected as a part of this protocol remains confidential.

Participants’ names, telephone numbers and email addresses are recorded by the clinical site staff at the time of consent to enable follow-up of the self-assessment questionnaire complementation and follow-up visits. Identifiable information is linked to stored data or samples only by a protected master list. This list is not shared outside the clinical site staff and no identifying information are transferred between study sites or to the study Sponsor.

All eCRF forms and samples are labelled only with a subject identification number and stored in suitable secure locations. Only persons who have undertaken the locally appropriate data protection training have access to the password-protected computer where entered data is stored.

The eCRF and the online questionnaire constitute the records held in the study main database. These will be retained for at least 25 years from the completion of the study. Identifiable data will be retained only as long as it is required. Informed consent forms will be retained securely by the recruiting sites depending on their jurisdictional requirements.

## Statistical methods

The statistical aspects of the study are summarised here with details fully described in a statistical analysis plan (SAP) currently being drawn up by the Statistical analyses group. The SAP will be finalised before any analysis takes place.

The SAP will be developed by the investigators and published whilst still blind to any analyses of aggregated data on study outcomes by treatment status. It will describe the outcomes, efficacy and safety variables and anticipated data transformations and manipulations, and other details of the analyses not provided in this study protocol.

General statistical methods are outlined hereafter.

### Statistical method

***Sample size.*** This is an observational study. We are interested in the effect of any available treatment against mpox used in the cohort and thus defined treatment outcomes (see below). However, due to the observational nature of this study, there is no formal sample size calculation to assess treatment effect. The availability of treatments and prescription rules differ in time and across countries. Any causal inference analysis will account for confounding effects, pre-specified in the statistical analytical plan.

***Participant flowchart.*** The total number of patients and the flowchart of the study will be presented with the number of patients excluded (with reasons for exclusion, and number of patients for each reason), included, receiving any antiviral drugs, lost-to-follow-up and analysed.

***Descriptive statistics.*** Patients’ demographics and baseline characteristics will be described overall and across analysis groups ( *e.g.*, treated with and without tecovirimat), by using, for continuous variables: sample size, mean, standard deviation, median, interquartile range, minimum and maximum; and for categorical variables: sample size, proportion and number of patients in different categories.

The number of missing values, overall and per group, will be reported.

***Inferential statistics.*** Causal inference methods will be used to derive information on the effects of tecovirimat (or other antiviral drugs) on patients’ outcomes (see below) and assessed from a Cox proportional hazard model. Graphics and tables associated to the analysis of each outcome will be fully detailed in the SAP.

***Primary outcome.*** The time to lesion resolution will be summarized for the overall population and stratified by treatment status using Kaplan-Meier curves, median time-to-event, proportion of resolution at D14, and associated 95% confidence intervals (CIs).

***Secondary outcomes.*** For clinical status at D14 and D28, counts and percentages will be presented with their associated 95% CI. The four-point ordinal scale will be used to estimate a proportional odds model. Recrudescence or relapse will be compared between treatment status with a logistic regression model.

Regarding virologic endpoints, the analysis will present the medians and interquartile ranges of mpox virus levels, measured in Cycle threshold (Ct) values, at different time points including baseline, and on days 4, 8, 14, and 28. These measurements will be used to assess viral clearance, categorized as a binary variable, in blood, skin lesions, and throat. The frequency of viral clearance will be reported based on the day of follow-up and treatment status groups.

Additionally, change in mpox virus DNA levels in throat and blood swabs will be compared to baseline on days 4, 8, 14, and 28 and will compared between treatment status using linear regressions at each timepoint.

Logistic regressions will model the presence of mpox virus DNA in lesion swabs at each timepoint.

Regarding safety endpoints, the number and type of SAEs, SARS and SUSARs within 28 days of enrolment will be described.

### Statistical methods

No method will be planned to account for multiplicity.

### Interim analyses

No interim analysis will be performed.

### Statistical methods

***Adjusted analyses.*** It will be specified in the SAP which confounders adjustments will be made for ( *e.g.*, age, pre-existing smallpox vaccination, extent of lesions, *etc.*).

### Statistical method

***Analysis population.*** All eligible participants will be included in the final analysis, *i.e.*, the final analysis set will be the overall population of enrolled participants (prospective and retrospective, with positive mpox PCR).

***Method to account for missing, unused or invalid data.*** The number, timing and pattern for missing values will be detailed for each outcome measure. For missing primary time-to-event outcome:

- The patients who are lost to follow-up or withdrew from the trial and who did not experience the event will be censored at their lost to follow-up or withdrawal date
- Patients who died before experiencing the event will be censored at day 14
- If the patient is hospitalized, the event will be censored at hospital discharge day, whereas if the patient is an outpatient, the event will be censored at the last available date in the medical assessment forms
- If the outcome is still missing, it will be searched in the Patient Self-Assessment Surveys (PSA)

For the four-point clinical scales at day 14 and day 28, missing data will be imputed by using the last observation carried forward (LOCF) method for “Death” and “All lesions are completely resolved and no serious complications” modalities. Missing outcomes will be searched as done for the primary endpoint.

Endpoints still missing after these recovery steps will remain missing in the analyses.

## Oversight and monitoring

### Composition roles, and responsibilities of the coordinating centres, and trial study committee

***Overall study coordination.*** University of Oxford, United Kingdom. Coordinating Centre (EU): ANRS|MIE; Coordinating Centre (Switzerland): Hôpitaux Universitaires de Genève; Coordination Centre (Singapore): Tan Tock Seng Hospital. Each coordinating centre is responsible for the management of the study in a specific country or region. The coordinating centres are responsible for arranging any study-specific agreements with the local participating sites. They also act as sub data processor and conduct on-site monitoring if necessary.

***Study Steering Committee (SSC).*** The role of the SSC is to provide overall supervision for the study on behalf of the Sponsor and to ensure that the study is conducted according to the protocol and all relevant regulations and local policies.

The responsibilities of the SSC are:

- To provide advice, through its Chair, to the Study Operations Committee (SOC) and the Sponsor on all aspects of the study
- To make suggestions for amendments and review and approve amendments to study documentation, including the study protocol, participant information sheets and case report forms (CRFs) provided by the SOC
- To approve the statistical analysis plan
- To ensure that all relevant approvals are obtained before the study begins
- To monitor and supervise the progress of the study towards its overall objectives, review accrual and results of the study, adherence to the protocol, patient safety and the consideration of new information of relevance to the study and the research question
- To assess integrity and completeness of data collected
- To consider new information relevant to the study *e.g.*, results from other studies that may have a bearing to the conduct of the study and deciding on appropriate action
- To provide input into the interpretation and writing up of the study results
- To maintain confidentiality of any study information that is not in the public domain
- To respond to study correspondence and any questions in a timely fashion

The MOSAIC Steering Committee is a multidisciplinary group who jointly have responsibility for the design, conduct and evaluation of the clinical research project.

***Study Operations Committee (SOC).*** The role of the Operations Committee is to oversee the day-to-day management, conduct and progress of the study. Any issues identified by the Operations Committee are reported to the Steering Committee.

The responsibilities of the SOC are as follows:

- To agree upon amendments to study documentation, including the study protocol, participant information sheets and case report forms (CRFs), and seek approval from the SSC before submission
- To finalise the statistical analysis plan for review by the SSC
- To finalise standard operating procedures (SOPs), the study monitoring plan and data monitoring plan (SSC approval not required)
- To provide clinical or other expert guidance on study-based matters such as clinical and practical queries and interpretation of information recorded on CRFs
- To oversee recruitment, address any issues with recruitment at study sites, and report any concerns to the SSC
- To oversee study monitoring and address any issues raised during centralised monitoring, including making recommendations for on-site monitoring, and report any concerns to the SSC
- To provide input into the interpretation and writing up of the study results
- To maintain confidentiality of any study information that is not in the public domain
- To respond to study correspondence and any questions in a timely fashion

The SOC should maintain confidentiality of all information it receives. Members should not discuss confidential issues from their involvement in the study until the primary results have been published.

The MOSAIC SOC is a multidisciplinary group who jointly have responsibility for the conduct of the clinical research project.

***The EU coordination Committee.*** The role of the EU coordination Committee is to oversee the day-to-day management, conduct and progress of the study in the EU participating countries.

***Virology Task force.*** The Virology task force group gathers virologists from the participating countries in the aim to share and harmonise virologic techniques for the study.

***Statistical analyses group.*** The Statistical analyses group develops and designs the Statistical Analysis Plan.

(see ‘Additional File 2: Appendix H’ in *Extended data* for Contributors’ names, roles and institutions) ^7^

### Composition of the data monitoring committee, its role and reporting structure

As this is not a randomised clinical trial, a data monitoring committee has not been convened. A medical monitor has instead been appointed to oversee issues related to participant safety.

The responsibilities of the medical monitor include, but are not limited to, commenting on any safety considerations related to the protocol, reviewing all Serious Adverse Event (SAE) reports and providing a causality assessment of the relationship between the event and tecovirimat and/or the study procedures, periodically reviewing a line listing of all AEs reported, reviewing all major protocol deviations, and providing advice on their management.

### Harms

***Safety reporting.*** The assessment of safety outcomes—a study objective—requires adverse event reporting, serious adverse event reporting and collection of pregnancy outcomes in both treated and non-treated cohorts.

The study procedures carry minimal risk and therefore AE related to the study procedures are not anticipated.

AE and SAE data are collected in the eCRF from the date that the participant’s diagnostic sample for mpox infection was collected or the date of enrolment (whichever comes first) until D28.

***Definition of Serious Adverse Events (SAE).*** The Serious Adverse Events (SAE) are defined as any unfavorable medical occurrence that meets one or more of the following criteria:

- Results in the participant's death.
- Poses a life-threatening risk to the participant's well-being.
- Requires hospitalization, including cases where it extends an ongoing hospital stay.
- Leads to persistent or significant disability or incapacity.
- Involves a congenital anomaly or birth defect.

Additionally, other "important medical events" may also be considered serious adverse events if, based on appropriate medical judgment, they have the potential to jeopardize the participant's health and necessitate medical or surgical intervention to prevent any of the outcomes mentioned above.

It is important to note that the term "life-threatening" within the definition of "serious" pertains to events where the participant was at risk of death at the time of the occurrence. It does not refer to events that hypothetically might have caused death if they were more severe.

All SAEs (including a site investigator causality assessment) should be reported by the site to the sponsor within 24 hours of the site becoming aware of the event and their causal relationship to tecovirimat (or other antivirals) are assessed. The Sponsor reviews all SAE reports received and conducts the causality and expectedness assessment of the event. Additional information (including the reason for considering the event both serious and related, and relevant medical and medication history) may be sought from the reporting site. The expectedness assessment considers Section 4.8 of the EMA Summary of Product Characteristics (SmPC) as Reference Safety Information for tecovirimat. Where possible, Section 4.8 of the relevant EMA SmPC is also used to assess the expectedness of the event for any other antiviral used in the study. The Sponsor conducts a causality and expectedness assessment within one business day of receiving the SAE report to determine whether the event is an SAE, SAR or SUSAR. In the event of divergent causality assessments by the site investigator and the Sponsor, both assessments are recorded and the assessment determining the strongest relationship defines the event as a SAE or SAR.

If there is a reasonable probability that any SAE is caused by tecovirimat or any other antiviral medication, it is considered a Suspected Serious Adverse Reaction (SSAR).

Participants who encounter a SAR or SSAR, which are events deemed to be associated with the treatment, will be followed up until the event is resolved.

All SUSARs are reported to:

- The EMA by the Sponsor’s Representative in the EU within seven days, if the event is fatal or life-threatening, or otherwise 15 days.
- The UK Research Ethics Committee (REC) within 15 days of becoming aware of the event
- The responsible ethics committee and SwissMedic in Switzerland within seven days, if the event is fatal, or within 15 days for all other events. *Note:* All SAEs resulting in death are reported to the ethics committee and SwissMedic within seven days.
- The Domain Specific Review Board (DSRB) by the Sponsor’s Representative in Singapore within seven days.

In countries where the study is considered an observational study, it is the responsibility of the treating clinician to report (serious) AE directly to national and regional regulatory bodies according to applicable national and regional legislation i.e., the MHRA’s Yellow Card system in the UK.

Please see ‘Appendix G, Additional File 2’ in *Extended data* for full details of the SAE reporting process for the study ^7^.

There may also be additional country specific requirements for safety reporting depending on regulatory requirements, in particular, the additional requirements for the EU/EEA are found in Appendix H, Additional File 2 “safety reporting for EU/EEA sites” section”.

### Frequency and plans for auditing trial conduct

No audit of the conduct of this programme is planned.

## Plans for communicating important protocol amendments to relevant parties ( *e.g.*, trial participants, ethical committees)

All amendments to the protocol are reviewed and approved by the responsible ethics committees before implementation.

## Dissemination policy

The results of this study are expected to be published in open-access peer-reviewed literature and presented at scientific conferences. A lay summary of the results will also be made available.

### Dissemination policy—Authorship eligibility guidelines

The Investigators will be involved in reviewing drafts of the manuscripts, abstracts, press releases and any other publications arising from the study. Authors will acknowledge that the study was funded by Wellcome, The Bill and Melinda Gates Foundation and for EU/EEA countries: MPX-Response. Authorship will be determined in accordance with the ICMJE guidelines and other contributors will be acknowledged.

## Dissemination policy—Public Access

A summary of the results will be made available in the publicly-facing EU CTIS platform within one year following the last visit of the last participant in the last participating EU country. A summary of the global results, if available later, will also be provided.

## Discussion

Observational studies are an integral part of a multi-pronged clinical research approach alongside interventional trials. They can rapidly generate solid evidence on clinical characterisation and outcomes, especially with a new emerging disease, such as the ongoing mpox clade IIb/III outbreak, and thus inform both practice and clinical trials design. However, in order for them to be successful, a number of criteria need to be fulfilled.

One is speed. MOSAIC got off to a fast start, recruiting the first patient in Switzerland on 4 July 2022, just two months after the start of the outbreak, but by October 2023 only six of the 10 participating countries have enrolled participants either prospectively or retrospectively. This is because most of the EU participating countries received approval only when the number of cases was already dropping and then faced contractual issues.

A detailed report of the various hurdles encountered has been shared with the European Medicines Agency (EMA), summarised in a paper for wider dissemination ^10^.

For evidence generation in health crises, we need a conducive regulatory and political environment, allowing shorter approval timelines and simplification of administrative regulation ^10^. Unfortunately, the MOSAIC experience shows that the system, at least in the European Union, is not ready. Countries where the study is considered as observational where able to start quickly (between, 13 and 20 days after the initial submission)——Switzerland enrolled the first case on 4 July 2023 after a two-week review process, quickly followed by France that had an initial approval as observational study, and then the UK. In comparison, in the other European Union participating countries, the median time to decision was 46.5 days (IQR 41 to 62) ^10^. An additional complication has been that tecovirimat—the only drug currently licensed for treating mpox in the EU and UK—has been available only in small quantities, and mostly through donations by the manufacturer, SIGA, when cases were peaking, and through a centralised purchase in the EU only much later (end of September 2022).

Beyond the operational aspects, starting clinical research for under-researched diseases like mpox is often slowed down by the lack of established methodologies for defining outcomes and endpoints to assess the disease and the treatment effect. MOSAIC benefitted from the tecovirimat Expanded Access Programme (EAP) protocol underway in the Central African Republic (CAR), which could be quickly adapted to the clade IIb ^6^, and from experience with clinical characterisation protocols. Outcomes have also further been harmonised across several other studies and trials currently running or planned on mpox: randomised clinical trials of Tecovirimat ( Platinum in the UK (ISRCTN17461766) and in Canada (NCT05534165), Unity in Switzerland, Brazil and Argentina (NCT05597735), Stomp (NCT05534984), EPOXI in Europe, PALM007 in Democratic Republic of Congo (NCT05559099), MOSA in Africa), EAPs (the US Army EAP (NCT02080767) and the Tecovirimat for mpox in Central African Republic (ISRCTN43307947)), the WHO Monitored Emergency Use of Unregistered and Investigational Interventions (MEURI) and observational studies (the Clinical Characterisation Protocol in UK and Netpox in Brazil (NCT05784038)). So that now hopefully consistent, comparable evidence will be generated, although some methodological questions are still unresolved ^11^. At the same time, uncertainties persist about outcomes and study endpoints, both clinical and virologic ^11^. For instance, current studies, including MOSAIC, tend define lesion resolution as “For skin lesions, typically this means the lesion has scabbed, desquamated and new layer of skin has been formed. For mucosal lesions, the phase of scabbing and desquamation is absent, and healing with new layer of skin ensues.” (UNITY trial) or “skin lesions are scabbed, desquamated or healed, and visible mucosal lesions are healed” (STOMP trial). However, there appear to be substantial inter-observer differences in lesion classification ^12^, and we know that the MPXV persists in scabs in both clade I and II cases ^13^

Then, beyond speedy set-up, there is also the need for sustainability, so that information can be accrued during protracted outbreaks, or such as ‘dormant’ protocols are maintained in-between outbreaks and can be quickly restarted as needed. Luckily, the current mpox outbreak has been curbed. At the time of writing, MOSAIC has enrolled 601 PCR-confirmed mpox cases of whom 55 treated with tecovirimat, 7 with cidofovir and 2 with both. The enrolment have been stopped and the last follow up visits are expected in January 2024.

## Study status

The current version of the protocol is version 2.0, dated 25 November 2022. The study was authorised for the first time in Switzerland on 22 June 2022, and is due to end on 31 January 2024.

## Declarations

### Research ethics approval

***Ethical and regulatory considerations.*** The Investigator ensures that this study is conducted in accordance with the principles of the Declaration of Helsinki.

***Approvals.*** Following Sponsor approval, the protocol, informed consent form, participant information sheet and any patient-facing materials material have been submitted to an appropriate Research Ethics Committee (REC) for each jurisdiction, and Health Research Authority (HRA) and host institutions for written approval.

The Investigator has submitted and, where necessary, obtained approval from the above parties for all substantial amendments to the original approved documents.

***Reporting.*** The Chief Investigator at the Sponsor or the Sponsor’s Representative (where applicable) shall submit once a year throughout the study, or on request, an Annual Progress report to the approving RECs, HRA (in the UK), host organisation, Sponsor and funder (where required). In addition, the start date of the clinical trial, inclusion date of the first subject, end of recruitment and end of the clinical trial for each member states and globally.

In addition, an End of Study notification and final report will be submitted to the same parties.

### Availability of data and materials

The final database will be shared with the statisticians of the University of Oxford.

Permission will be sought from the responsible ethics committees to make pseudonymised individual patient data available for secondary analysis.

## Data Availability

No data are associated with this article. Figshare: MOSAIC A cohort study of human mpox virus disease. https://doi.org/10.6084/m9.figshare.23545158.v1 ^7^. This project contains the following underlying data: **Additional file 1** – SPIRIT checklist **Additional file 2** - Appendixes to the study protocol for a cohort study of human mpox virus disease (MOSAIC) Data are available under the terms of the Creative Commons Attribution 4.0 International license (CC-BY 4.0).
