# Supplementary material for: MOSAIC: A cohort study of human mpox virus disease
Source: Wellcome Open Res. 2023 Dec 12;8:415. Originally published 2023 Sep 19. [Version 3] doi: 10.12688/wellcomeopenres.19616.3 (PMC10685065; doi:10.12688/wellcomeopenres.19616.3)
Supplement: Peer review history

# Reviewer report: Reviewer response for version 2

**DOI:** 10.21956/wellcomeopenres.22483.r69854

**Published:** 2023-11-28

**Authors:** Yon C. Yu

**Affiliations:** 1 Medical Countermeasures Regulatory Support Unit, Office of Readiness and Response, Centers for Disease Control and Prevention, Atlanta, Georgia, USA

While the purpose of MOSAIC study is to identify and characterize clinical and virologic outcomes of laboratory-confirmed mpox patients, the focus of this article, however, is on describing the study protocol itself with implementation process and operational challenges. Given this, suggest consideration for adding a similar text as in the Introduction section (".. describe the design of the ongoing MOSAIC study..") to the Background in the Abstract for clarity. Overall, the MOSIAC study protocol, designed as an observation cohort study, is well described and there are no major concerns. Below are a few suggestions for consideration. 

- Regarding the inter-observer differences mentioned, please consider commenting on any specific measures/coordinating efforts taken (or to suggest in retrospect) to reduce the subjective variability with lesion assessment (e.g., through SOPs, definitions/refining in eCRF instructions, adjudication process, etc) that may be useful to future study considerations. 
- Regarding recruitment efforts, please consider commenting on any active, targeted communications and socializing the study information to at-risk groups for mpox to help increase study participation.
- With 601 PCR-confirmed mpox patients enrolled, of which 66 treated with antiviral(s), suspecting that data analysis and sub-analysis plans likely will evaluate any differences in clinical and virologic outcomes by co-variates (e.g., age, gender, race, ethnicity, immunocompromising conditions) within respective untreated and treated groups and between untreated and treated patients to the extent possible, depending on matching variables applied. The results of MOSAIC study will be of interest and recognize the mention that a statistical analysis plan is being finalized. While the anticipated summary of results is pending, it may be worth including a reference the poster presentation that reported on the initial 122 mpox cases.   

Is the study design appropriate for the research question?

Yes

Is the rationale for, and objectives of, the study clearly described?

Yes

Are sufficient details of the methods provided to allow replication by others?

Yes

Are the datasets clearly presented in a useable and accessible format?

Not applicable

Reviewer Expertise:

Scientific regulatory expertise with medical countermeasures (MCMs) for high consequence threats or emerging infectious diseases; MCM preparedness and response

I confirm that I have read this submission and believe that I have an appropriate level of expertise to confirm that it is of an acceptable scientific standard.

# Reviewer report: Reviewer response for version 2

**DOI:** 10.21956/wellcomeopenres.22483.r69371

**Published:** 2023-10-31

**Authors:** Naman Shah

**Affiliations:** 1 Medical and Dental Affairs, Los Angeles County Department of Public Health, Los Angeles, CA, USA

The authors made appropriate edits and addressed all my concerns. The article should be approved.

Is the study design appropriate for the research question?

Yes

Is the rationale for, and objectives of, the study clearly described?

Yes

Are sufficient details of the methods provided to allow replication by others?

Yes

Are the datasets clearly presented in a useable and accessible format?

Not applicable

Reviewer Expertise:

NA

I confirm that I have read this submission and believe that I have an appropriate level of expertise to confirm that it is of an acceptable scientific standard.

# Reviewer report: Reviewer response for version 1

**DOI:** 10.21956/wellcomeopenres.21730.r67712

**Published:** 2023-10-13

**Authors:** Naman Shah

**Affiliations:** 1 Medical and Dental Affairs, Los Angeles County Department of Public Health, Los Angeles, CA, USA

This is a description of the MOSAIC study protocol, progress and challenges. MOSAIC is an observational cohort of mpox patients that seeks to describe the clinical outcomes irrespective of treatments received. 

 The protocol is well designed and there's a great discussion on the challenges of endpoints, recruitment, and multicounty logistics when working on outbreaks of neglected diseases. The use of SPIRIT guidelines enhances the quality of the protocol and its reporting as well. 

 Major issues

- Of the three goals in the paper, 90% of the effort is on describing the protocol, 9% on challenges encountered, and 1% on progress. I think this would be a stronger paper if information shared on progress was expanded. For example, not just progress in enrollment, but why have only 5 countries enrolled any participants? Progress in retention/follow-up, progress in outcome assessment (given the challenges with endpoints), etc. The progress section should also precede challenges rather than be at the end. 

Minor issues

- From my own clinical experience, I am concerned with scabbed lesions as an endpoint. Scabs are infectious and so are not a great public health endpoint, and in some patients, e.g. AIDS, scabs have remained non-healing for months, are large, and depending on wound care can be dry or exudative with surrounding edema, and thus as a patient facing outcome not represent meaningful resolution either. 

Is the study design appropriate for the research question?

Yes

Is the rationale for, and objectives of, the study clearly described?

Yes

Are sufficient details of the methods provided to allow replication by others?

Yes

Are the datasets clearly presented in a useable and accessible format?

Not applicable

Reviewer Expertise:

Infectious disease epidemiology

I confirm that I have read this submission and believe that I have an appropriate level of expertise to confirm that it is of an acceptable scientific standard, however I have significant reservations, as outlined above.

# Response

**Published:** 2023-10-20

**Authors:** ELISE PESONEL

**Affiliations:** ISARIC - PSI, University of Oxford, Oxford, England, UK

**RESPONSE TO MAJOR ISSUES:**

 The aim of this article is to provide public access to our protocol, thereby informing the scientific community about the conduct and methodology behind our ongoing Mpox research and ensure transparency when reporting of our results. Additionally, we have published another paper addressing the challenges encountered in implementing the study, with a specific focus on the European regulation : Patrick-Brown TD, Bourner J, Kali S, et al. Experiences and challenges with the new European Clinical Trials Regulation. Research Square; 2023. DOI: 10.21203/rs.3.rs-2514582/v1.. As for progress, we have enrolled 601 confirmed cases. While the enrolments have been stopped and the last follow-up visits are expected in January 2024, we are currently analysing the data on the primary endpoints and working towards publishing the results as soon as possible. We have modified the article accordingly.  

**RESPONSE TO MINOR ISSUES**: We agree that scabs can be PCR-positive and a source of transmission – for clade I mpox, please also see our paper Garba-Ouangole S, Bourner J, Mbrenga F, Gonofio E, Selekon B, Manirakiza A, Kalthan E, Malaka C, Boum Y 2nd, Olliaro P, Nakouné E. Laboratory Diagnosis of Mpox, Central African Republic, 2016-2022. Emerg Infect Dis. 2023 Sep;29(9):1846-1849. The endpoint has been aligned with other protocols that have started around the same time to allow comparability – see e.g. Assessment of the Efficacy and Safety of Tecovirimat in Patients With Monkeypox Virus Disease - Full Text View - ClinicalTrials.gov Study of Tecovirimat for Human Monkeypox Virus - Full Text View - ClinicalTrials.gov Antiviral treatment with tecovirimat for patients managed at home with monkeypox We agree this is far than ideal and have further elaborated on this in the paper
